# *Taenia solium*: Development of an Experimental Model of Porcine Neurocysticercosis

**DOI:** 10.1371/journal.pntd.0003980

**Published:** 2015-08-07

**Authors:** Agnès Fleury, Armando Trejo, Humberto Cisneros, Roberto García-Navarrete, Nelly Villalobos, Marisela Hernández, Juana Villeda Hernández, Beatriz Hernández, Gabriela Rosas, Raul J. Bobes, Aline S. de Aluja, Edda Sciutto, Gladis Fragoso

**Affiliations:** 1 Unidad Periférica del Instituto de Investigaciones Biomédicas en el Instituto Nacional de Neurología y Neurocirugía, Universidad Nacional Autónoma de México, México D.F., México; 2 Instituto Nacional de Neurología y Neurocirugía, Secretaría de Salud, México D.F., México; 3 Facultad de Medicina Veterinaria y Zootecnia, Universidad Nacional Autónoma de México, México D.F., México; 4 Instituto Nacional de Pediatría, Secretaría de Salud, México D.F., México; 5 Hospital General Naval de Alta Especialidad, Secretaría de Marina (SEMAR), México D.F., México; 6 Departamento de Inmunología, Instituto de Investigaciones Biomédicas, Universidad Nacional Autónoma de México, México D.F., México; 7 Facultad de Medicina, Universidad Nacional Autónoma de México, México D.F., México; 8 Facultad de Medicina, Universidad Autónoma del Estado de Morelos, Morelos, México; Universidad Peruana Cayetano Heredia, PERU

## Abstract

Human neurocysticercosis (NC) is caused by the establishment of *Taenia solium* larvae in the central nervous system. NC is a severe disease still affecting the population in developing countries of Latin America, Asia, and Africa. While great improvements have been made on NC diagnosis, treatment, and prevention, the management of patients affected by extraparenchymal parasites remains a challenge. The development of a *T*. *solium* NC experimental model in pigs that will allow the evaluation of new therapeutic alternatives is herein presented. Activated oncospheres (either 500 or 1000) were surgically implanted in the cerebral subarachnoid space of piglets. The clinical status and the level of serum antibodies in the animals were evaluated for a 4-month period after implantation. The animals were sacrificed, cysticerci were counted during necropsy, and both the macroscopic and microscopic characteristics of cysts were described. Based on the number of established cysticerci, infection efficiency ranged from 3.6% (1000 oncospheres) to 5.4% (500 oncospheres). Most parasites were caseous or calcified (38/63, 60.3%) and were surrounded by an exacerbated inflammatory response with lymphocyte infiltration and increased inflammatory markers. The infection elicited specific antibodies but no neurological signs. This novel experimental model of NC provides a useful tool to evaluate new cysticidal and anti-inflammatory approaches and it should improve the management of severe NC patients, refractory to the current treatments.

## Author Summary

Neurocysticercosis (NC) is caused by the implantation of the larval stage of *Taenia solium* in the human central nervous system. Although NC diagnosis, treatment, and prevention have clearly improved in the last 40 years, the disease still causes significant morbidity and mortality in endemic regions of Latin America, Asia, and Africa. In industrialized countries, the number of diagnosed cases has increased in recent years due to immigration. In this paper, we introduce a new experimental model of *T*. *solium* neurocysticercosis in pigs. Activated oncospheres were surgically implanted in the subarachnoid space of the cerebral convexity in piglets. Then, the animals were observed during 4 months. An increase in anti-cysticercal antibodies was detected, along with an inflammatory reaction surrounding the established parasites. This experimental model of *T*. *solium* NC will improve our knowledge on the pathogenesis of the disease; additionally, it will let us evaluate new promising treatments for inflammation and improve the effectiveness of cysticidal drugs.

## Introduction

The larval stage of *Taenia solium* can establish itself in different tissues of swine and human hosts after they ingest *T*. *solium* viable eggs [1]. The adult intestinal tapeworm develops when humans consume cysticercus-infected, improperly cooked pork meat. The adult worm produces millions of eggs, which are released to the environment by the host in feces and may contaminate the water, soil, and food [2]. Endemicity is clearly related to poor hygienic standards and sanitary conditions; i.e., absence or inadequate use of latrines, open-air defecation, traditional pig farming, lack of meat inspection, inadequate water supply, and lack of drainage [2,3]. These conditions prevail in developing countries of Latin America, Asia, and Africa, where cysticercosis is endemic and poses a major health and economic challenge [4,5]. Recently, the World Health Organization (WHO), the Food and Agriculture Organization (FAO), and the UK Department for International Development (DFID) listed *T*. *solium* infection as one of the 17 neglected zoonotic diseases that can be effectively controlled [6].

In humans, the metacestode frequently establishes in the central nervous system, causing neurocysticercosis (NC), the most severe form of the disease [1]. In pigs, cysticerci are usually found both in muscle tissue and in the brain [2].

One of the main challenges in human NC is the low efficacy of anti-cysticidal and anti-inflammatory treatment when cysts are located in the subarachnoid or ventricular spaces. Frequently, anti-cysticidal drugs (albendazole and praziquantel) are only partly effective in these extraparenchymal NC forms [7–9]. Moreover, the neuroinflammation that accompanies these NC forms frequently results in arachnoiditis and vasculitis, which increase the disease severity. Currently, corticosteroids are given to NC patients to control neuroinflammation [10]. However, the administration of high corticosteroid doses administered for long periods to control neuroinflammation frequently promotes severe peripheral side effects, like steroid-induced diabetes [11]. This situation points to the need of investigating the effectiveness of other cysticidal drugs and more specific anti-inflammatory drugs to treat these patients.

In this regard, a suitable experimental model for cysticercosis will be a useful tool to search and evaluate new therapeutic options. Several experimental models have been used to study cysticercosis. An artificial infection caused by the inoculation of *T*. *crassiceps* cysticerci (ORF strain) into the abdominal cavity of mice has been the most extensively used one. This model has contributed to our understanding of the impact of immune, sexual, genetic, endocrine, and behavioral factors on the infection [12–15]. The model was also employed to test promising antigens for vaccination against *T*. *solium*, based on the cross-reactivity between *T*. *crassiceps* and *T*. *solium* antigens [16,17]. However, the intraperitoneal environment in this experimental model hardly resembles the conditions prevailing in the central nervous system. Two recent reports of intracerebral infection with *Taenia crassiceps* offer hope on its potential to evaluate NC treatments [18,19]. A murine intracerebral infection with *Mesocestoides corti* was also developed [20,21]. Nevertheless, any extrapolation of the results obtained in those intracerebral models should be made with caution, due to the differences between these cestodes and *T*. *solium*.

With respect to porcine cysticercosis, a *T*. *solium* intramuscular model has been developed, but it does not allow studying NC [22]. On the other side, orally infected pigs have been used in some studies [23–25]; unfortunately, infection rates are low and variable, particularly in brain tissue, preventing its use to study the response of brain cysticerci to treatment. Naturally infected pigs were also used in some studies, evaluating the cerebral infection by Magnetic Resonance Imaging (MRI) [26]. While this approach is interesting, access to MRI in endemic countries is restricted even for humans, and MRI studies in pigs are not feasible.

Considering the limitations of the available experimental models, the results obtained in the development of a porcine NC model are herein presented.

## Material and Methods

### Ethics Statement

This study was approved by the Institutional Animal Care and Use Committees of the Facultad de Medicina Veterinaria y Zootecnia (FMVZ), UNAM and of the Instituto Nacional de Neurología y Neurocirugía, Mexico. All guidelines in the Official Mexican Norm (NOM-062-ZOO-1999) on the technical specifications for the production, care, and use of laboratory animals were followed.

The adults that were treated to find the pork tapeworm (*Taenia solium*) provided written informed consent. In cases of minors, written informed consent from the person in charge of the minor was also required before any intervention.

### Pigs

Twenty-four 2-month-old crossbred York-Landrace piglets of different sexes were purchased from a technically operated, cysticercosis-free farm, and then transferred to the FMVZ, UNAM, to be employed in the two experiments reported in this study. All animals were bled before infection and every 20 days after infection until sacrifice; sera were separated and frozen until used for immunological tests.

### Parasites

Adult *Taenia solium* worms were retrieved from human patients living in Mexican rural endemic areas, who reported proglottid expulsion.

Oral treatment with niclosamide (Bayer, S.A., Mexico) in a single 2-g dose, followed by intestinal purge one hour after, was administered. Once obtained, adult tapeworm specimens were macro- and microscopically inspected to distinguish between *T*. *solium* and *T*. *saginata*, based on morphological characteristics. Species was confirmed by PCR using a previously described procedure [27].

Afterwards, *T*. *solium* proglottids were washed three times with physiological saline solution and maintained in PSS (sodium polystyrene sulfonate) and PBS (phosphate-buffered saline) with antibiotics (penicillin-streptomycin) at 4°C until use. Parasites were conserved under refrigeration for 4 weeks in the first experiment and for 7 days in the second one.

### Hatching and Activation

A few hours before surgery, *in vitro* egg hatching was performed under sterile conditions using 0.75% sodium hypochlorite in water as previously reported [28]. After extensive washing with PBS, subsequent oncosphere activation was slightly different in the two experiments. In the first one, RPMI-1640 added with 10% trypsin and 5% pig bile was used, while artificial intestinal fluid with 1% pancreatin, 0.2% anhydrous sodium carbonate, 10% trypsin and 0.5% pig bile in RPMI-1640 (Gibco) was used in the second one. In both trials, oncospheres were incubated in a water bath for 1 h at 37°C, shaking the oncospheres every 15 minutes. At the end of incubation, activated oncospheres (those with detectable movement) were counted and their viability was assessed using trypan blue (Sigma). Oncosphere activation and viability was about 20% and 85% for the first and the second experiment, respectively. For surgical implantation, activated oncospheres were thoroughly washed with sterile saline solution to eliminate all enzyme content.

### Surgical Implantation and Follow-Up

Animals were anesthetized by intramuscular administration of xylazine (2.2 mg/kg), ketamine (2.2 mg/kg), and Tiletamine-Zolacepam (4.4 mg/kg) followed by isoflurane. Craniotomy was performed for subarachnoid and ventricular oncosphere implantation. Craniotomy was centered at the intersection planes located 2 cm above the external auditory canal in the coronal plane and 2 cm lateral to the midline. For subarachnoid implantation, sulcus dissection was done and oncospheres were implanted at the deep sulcus surface. For ventricular implantation, oncospheres were delivered via direct puncture at the frontal horn of lateral ventricle with a latex catheter (internal diameter 6 Fr). The volume of sterilized saline solution inoculated was 100 μl for 500 oncospheres and 200 μl for 1000 oncospheres.

In the first experiment, seven piglets were inoculated in the subarachnoid space; five received a high dose of oncospheres (1000–1500) and two received a low dose (100–150). Five animals were inoculated via ventricle, two with a high dose and three with a low dose. In the second experiment, eight piglets were inoculated in the subarachnoid space, four with 500 oncospheres and four with 1000 oncospheres. As controls, two pigs were inoculated in the muscle of the right rear leg, one with 500 and one with 1000 oncospheres, and two animals were operated but not inoculated (sham controls).

After surgery, all animals were kept in the facilities of FMVZ, UNAM for 4 months. Clinical status was checked daily, and blood samples were collected every 20 days by puncture of the anterior vena cava. Samples of cerebrospinal fluid (CSF) were also retrieved during surgery and at necropsy.

### Levels of Anti-cysticercal Antibodies and HP10 Secretion Antigen

Serum samples from subject pigs were collected before and after infection to measure specific IgG antibodies and the HP10 secretion antigen by ELISA. All samples were run in duplicate.

Anti-cysticercal antibodies and the HP10 antigen were detected by ELISA, as previously described [29]. Briefly, for antibody detection, polycarbonate Immulon I plates (Nunc, Roskilde, Denmark) were sensitized with 1 μg/well of *T*. *solium* cysticercal antigens (TsAg) in carbonate buffered saline, pH = 9.6 overnight at 4°C. The plate was washed and blocked with 200 μl PBS containing 1% w/v bovine serum albumin and 0.3% v/v Tween 20 and left for 60 min at 37°C. Serum samples were diluted 1:100 and the reaction was detected with 100 μl/well of HRP-goat anti-pig IgG (Fc) (Serotec) diluted 1:60,000. The reaction was developed with 100 μl/well of tetramethylbenzidine (TMB) (Zymed, San Francisco, California, USA) for 11 min at 4°C in the dark and stopped by adding 100 μl 0.2 M H_2_SO_4_ (Baker, Estado de Mexico, Mexico). OD values were measured at 450 nm in an ELISA reader (Opsys MR Dynex Technology, Chantilly, Virginia, USA).

For HP10 antigen detection, Immulon I plates (Nunc, Roskilde, Denmark) were sensitized with 1 μg/well of McAb HP10 diluted in 0.07 M saline buffered with 0.1 M borate, pH = 8.2, overnight at 4°C. Plates were washed four times with 200 μl/well of 0.15 M saline containing 0.05% v/v Tween 20; then they were blocked with 200 μl/well of PBS containing bovine serum albumin 1% w/v and 0.05% v/v Tween 20, and left for 60 min at room temperature. Later, 100 μl/well of undiluted serum samples were added and incubated for 30 min at 37°C, followed by incubation with biotinylated McAb HP10 diluted 1:500 for 30 min at 37°C and 100 μl/well of streptavidin-peroxidase conjugated (Amersham Ltd), diluted 1:4000 and incubated in the same conditions. The reaction was developed by adding 100 μl/well of tetramethylbenzidine (TMB) (Zymed, San Francisco, California, USA) for 30 min at 4°C in the dark and stopped with 100 μl/well of 0.2 M H_2_SO_4_ (Baker, Estado de Mexico, Mexico). OD values were measured at 450 nm in an ELISA reader (Opsys MR Dynex Technology, Chantilly, Virginia, USA).

A sample was considered as positive for HP10 and anti-cysticercal antibodies if the mean OD value at 450 nm was higher than the cut-off value, which was set based on the mean OD plus 2 SD in serum before infection. Cut-off for antibodies was 0.3, while cut-off for HP10 was 0.22.

### Necropsy

Four months after infection, the pigs were humanely killed and all tissues were inspected. The brains were extracted and macroscopic examinations were performed to detect external parasites. Afterwards, the brains were sliced for histological studies [30]. Parasites were regarded as vesicular when cyst membranes were thin and the liquid content was clear. In the colloidal stage, cyst membranes thicken and the liquid within the cyst turns opaque.

The muscles where cysticerci were inoculated were also inspected, and the number and degenerative stage of cysticerci were registered.

### Histological Studies

Tissue samples taken at necropsy were fixed in Zamboni solution (1.6% [w/v] paraformaldehyde, 19 mM KH_2_PO_4_ plus 100 mM Na_2_HPO_4_·7H_2_O in 240 ml of saturated picric acid and 1600 ml H_2_O), embedded in paraffin and stained with hematoxylin-eosin. Fifteen days after, 0.5-μm slides were prepared and microscopically observed to identify and evaluate the size, location, and degenerative stage of cysticerci. The degree of inflammatory reaction surrounding each cyst was evaluated according to the scale by Vargas and de Aluja, 1988 [30]. Briefly, this classification describes seven inflammation grades according to the cellular characteristics of the tissue surrounding parasites and the traits of parasites themselves. Grade 0: No inflammatory reaction. Grade 1: Discrete focal infiltration, mainly of lymphocytes, plasma cells, and eosinophils. Grade 2: Increase of infiltration, predominating lymphocytes and plasma cells; eosinophils are numerous and macrophages appear. Grade 3: The same cell populations are present; eosinophils adhere to the vesicular wall of the parasite, and the tegument becomes swollen and vacuoles appear within; macrophages begin to line up in a palisade pattern. Grade 4: The inflammatory reaction surrounds completely the parasite and the aggregates of lymphoid cells are larger; the capsular tegument shows marked degeneration and the bladder cavity is filled with acidophilic material and necrotic cells. Grade 5: The parasite is completely degenerated; lymphocytes, plasma cells and eosinophils are less frequent. Grade 6: Inflammatory cells are scarce.

### Immunohistochemical Analysis

Brains were fixed in Zamboni solution for at least one week. Afterwards, specimens were dehydrated and embedded in paraffin, and 5-μm sections were cut. Endogenous peroxidase was inhibited by incubation with 0.3% (v/v) H_2_O_2_ in PBS for 10 min. After washing twice with PBS, heat-mediated antigen retrieval method was performed by microwave treatment with 0.1 M sodium citrate solution (pH 6.0) for 5 min. Then, slides were rinsed three times in PBS buffer and sections were preincubated in a blocking solution consisting of 2% BSA (bovine serum albumin; Sigma-Aldrich) for 30 min. After two washes with Tris-EDTA buffer, sections were incubated with the primary antibody (described below) diluted in PBS buffer overnight at 4°C. After washing three times in PBS/A-T (1% BSA in PBS, plus 0.1% Triton X-100), 5 min each, slides were covered with secondary antibody conjugated with horseradish peroxidase (Dako-Kit) for 30 min at 37°C and rinsed with PBS/A-T. Peroxidase activity was visualized by incubating the samples for 2 min with 3-diaminobenzidine tetrahydrochloride (DAB, DAKO). Reaction was stopped with water, and sections were counterstained with hematoxylin, dehydrated, cleared, and mounted with permount (Fisher Scientific). The single labeled sections were examined by light microscopy Leica Galen III, and digital color video camera (SSC-DC14), on a Pentium IV, Windows 2000 computer.

The primary antibodies used in this study recognized: glial fibrillary acidic protein (GFAP; polyclonal rabbit DAKO, Glostrup, Denmark 1:100 dilution), vimentin (mouse clone V9, DAKO; 1:100 dilution), neuronal nuclear protein (NeuN; mouse clone MAB377, IgG; Chemicon, Temecula CA, USA; 1:1000), nestin (mouse anti-nestin monoclonal antibody, 1:100; Chemicon, Millipore Billerica, MA, USA), IL-4 (anti-human monoclonal antibody, 1:200 dilution, Biolegend), IL-6 (anti-human monoclonal antibody, 1:250 dilution Biolegend), IL-10 (anti-human monoclonal antibody, 1:100, Biolegend), IL-17A (anti-human monoclonal antibody, 1:150 Boise’s), TNF-α (anti-human monoclonal antibody, 1:500, Biolegend), CD54 (anti-human monoclonal antibody, 1:200 Biolegend), CD69 (anti-human monoclonal antibody, 1:200 Biolegend), CD80 (anti-human monoclonal antibody, 1:300 Biolegend), CD106 (anti-human monoclonal antibody, 1:250, Biolegend).

Anti-inflammatory (IL-4), immunoregulatory (IL-10), and proinflammatory (IL-6, IL-17A, TNF-α) cytokines were evaluated. CD54 was used to assess the expression of intercellular adhesion molecule-1 (ICAM1); CD69 was used to evaluate the activation of T lymphocytes; CD80 was used to evaluate the activation of B lymphocytes; and CD106 was used to evaluate the expression of the vascular cell adhesion protein-1 (VCAM1). The expression of glial fibrillary acidic protein (GFAP) and vimentin evaluates the activation status of astrocytes; nestin expression indicates the presence of immature neurons; and NeuN expression shows the presence of viable neurons.

Brain sections from distal areas to the parasite location or brain sections from sham-operated pigs were used as control.

### Statistical Analysis

Statistical analysis was performed with the SPSS software. Student’s *t*-test was used to evaluate statistical differences between means. Linear regression was used to test changes in OD values between sampling times.

## Results

### First Experiment

One pig from the group inoculated with a higher dose of oncospheres in the subarachnoid space showed fever and ataxia 20 days after the challenge. In spite of the treatment with antibiotics and anti-inflammatory drugs, its neurological status worsened and it was killed 30 days after infection. An abscess and a vesicle of 0.2 cm × 0.05 cm in size were found in the brain by macroscopic examination. Under microscopic examination, the vesicle was confirmed as a vesicular cysticercus surrounded by inflammatory reaction consisting of lymphocytes, plasma cells, and macrophages.

No other animal in this first experiment showed any neurological sign until sacrifice, 4 months after infection. A single parasite was observed in two pigs, both of which were inoculated with a higher dose of oncospheres in the ventricular system. In the first case, the cyst was located in the subarachnoid sulcus of the parietal lobe, near to the cortex. It was a vesicular cyst, 0.5 cm in diameter; it presented a scolex, as confirmed by microscopic examination. A grade-4 inflammatory reaction was observed around the cyst. In the second case, a colloidal cyst was located in the subarachnoid space of the convexity, in the left hemisphere near the interhemispheric fissure. It was 0.4 cm in diameter and it was surrounded by a grade-4 inflammatory reaction. No parasite or inflammatory reaction was detected in any other animal.

### Second Experiment

**Fig 1:**
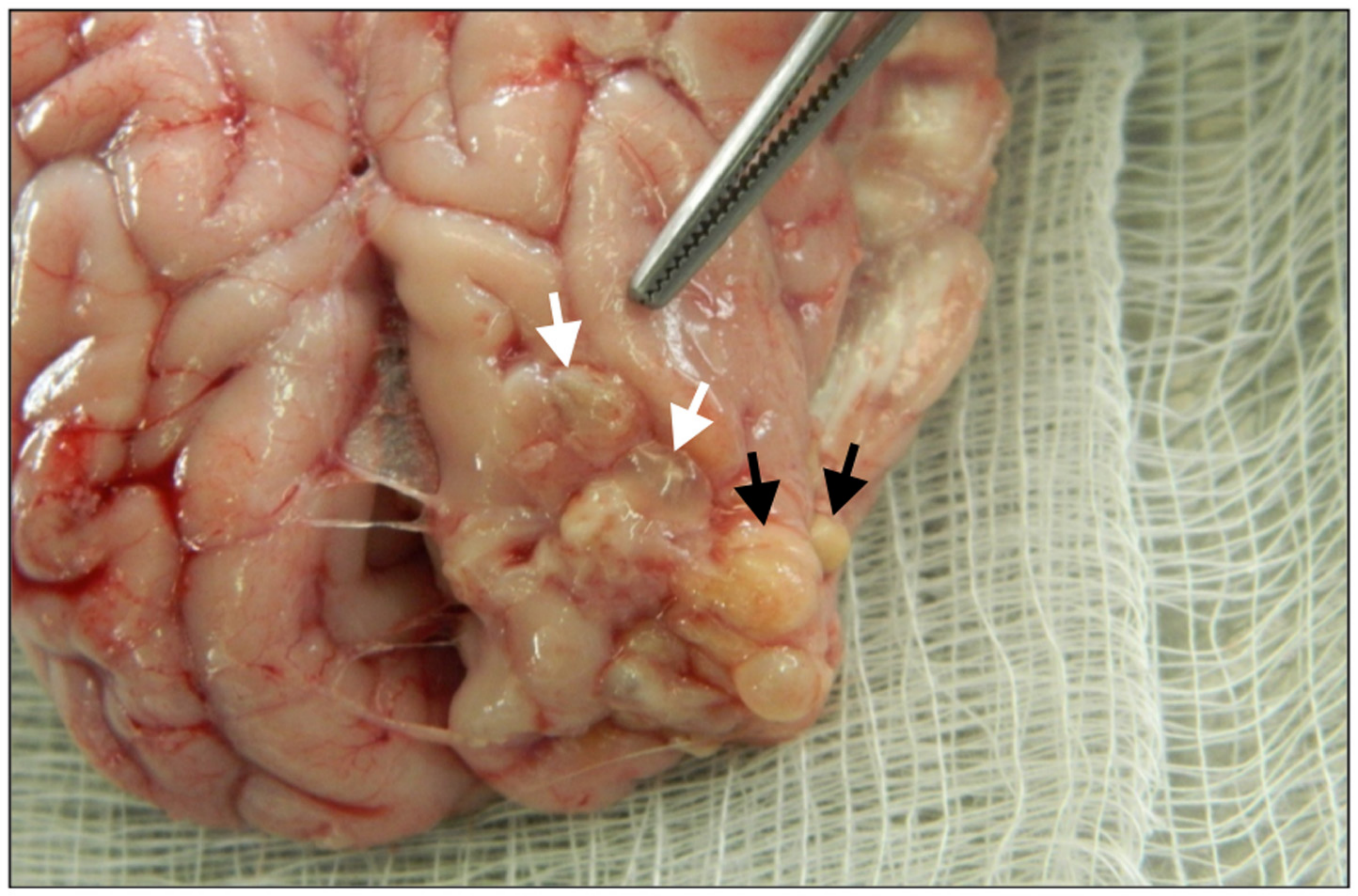
Macroscopic aspect of cysticerci recovered from brain. Vesicular (white arrows) and colloidal/caseous (black arrows) parasites are shown.

**Fig 2:**
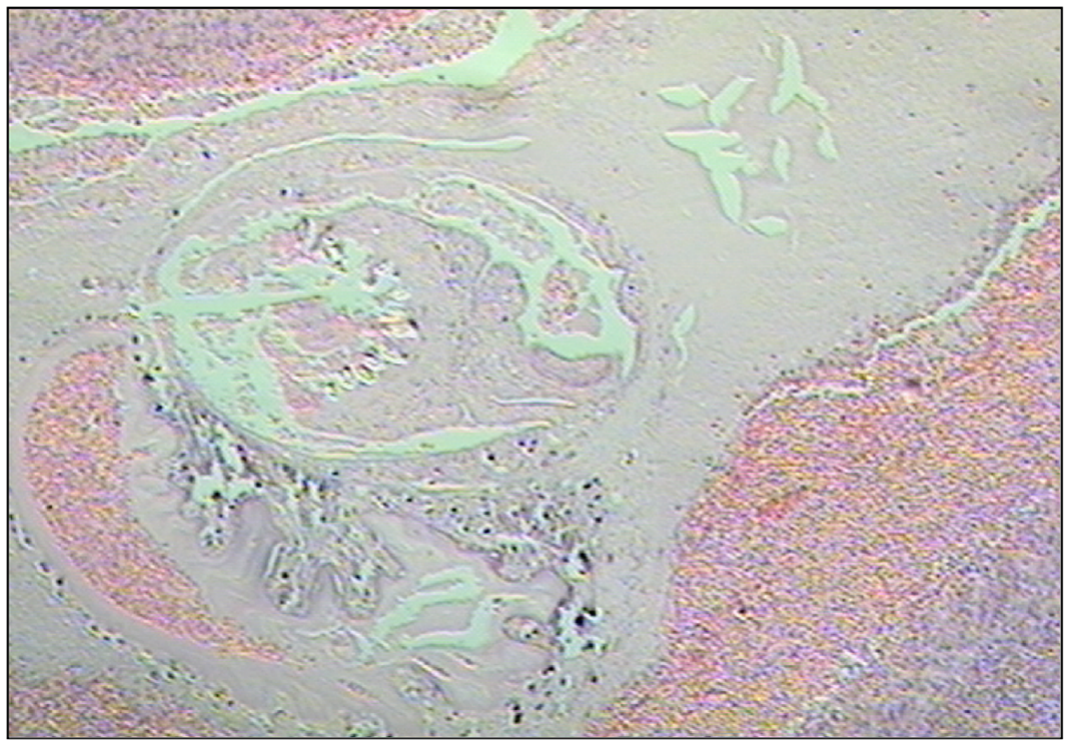
Microscopic examination (HE, 4x) of a cysticercus recovered from brain. Lymphocyte Aggregates are observed in the parasite periphery. Parasite structures are not visible. A grade-4 inflammatory reaction is shown.

During this experiment and until sacrifice, pigs were in good health condition, with no neurological signs. Macro- and microscopic examination revealed the presence of cysticerci in all animals. Cysts were located either in the subarachnoid space when the infection was induced in this compartment or in muscles when oncospheres were intramuscularly inoculated (Figs 1 and 2). All parasites were located in the proximity of the site of infection.

As shown in Table 1, 63 cysticerci from the brain and 11 cysticerci from the muscle were macroscopically examined. The number of cysts in the brain was higher when 1000 oncospheres were inoculated than those recovered when 500 oncospheres were used (36 versus 27). The efficiency of infection (number of developed cysticerci / number of oncospheres inoculated) was higher when lower doses of oncospheres were inoculated (3.6% with 1000 oncospheres versus 5.4% with 500 oncospheres, *P* = 0.1). The proportion of vesicular parasites was higher when higher doses of oncospheres were inoculated (33.3% (9/27) with 500 oncospheres versus 44.4% (16/36) with 1000 oncospheres), but the difference was not statistically significant (*P* = 0.44). The number of cysticerci found in the muscle was similar (5 versus 6), disregarding the dose of oncospheres inoculated; all cysts recovered from the muscle were in colloidal/caseous stage. The difference in the proportion of vesicular cysticerci found in brain (34%) and muscle (0%) was significant (*P* = 0.01).

**Table 1:** Macroscopic examination of cysticerci recovered after implantation of activated oncospheres in brain and muscle (Experiment 2).

Location and number of oncospheres	Pig No.	Number of developed cysticerci	Developmental stage of cysticerci	
			Vesicular	Co/Cs
SA, 500 oncospheres	1	7	2	5
	2	2	2	0
	3	3	0	3
	4	15	5	10
	**Total**	**27**	**9**	**18**
SA, 1000 oncospheres	5	11	8	3
	6	11	4	7
	7	10	3	7
	8	4	1	3
	**Total**	**36**	**16**	**20**
IM, 500 oncospheres	9	6	0	6
IM, 1000 oncospheres	10	5	0	5
**Total cysticerci**		**74**	**25**	**49**

SA: subarachnoid. IM: intramuscular. Co/Cs: colloidal/caseous.

The results of the histopathological examination of brain tissue samples and parasites are shown in Table 2 (Fig 2). Most cysticerci were in a degenerating stage and were surrounded by an exacerbated inflammatory reaction (grade 4). No difference was observed in the inflammatory grade in the two groups of pigs (500 versus 1000 oncospheres inoculated). When comparing grade-2 and grade-3 inflammation versus grade-4 and grade-5 inflammation, 33.3% of animals (8/24) in the 500 oncospheres group versus 34.6% of animals (9/26) in the 1000 oncospheres group showed a low inflammatory reaction (grades-2,3; *P* = 1).

**Table 2:** Histopathological classification of the inflammatory reaction (Experiment 2).

Location and number of oncospheres	Pigs	Histopathological grade							
		0	1	2	3	4	5	6	Total
**SA, 500 oncospheres**	1	-	-	-	-	5	-	-	5
	2	-	-	-	-	1	-	-	1
	3	-	-	-	3	-	-	-	3
	4	-	-	-	5	10	-	-	15
	**Total**				**8**	**16**			**24**
**SA, 1000 oncospheres**	5	-	-	-	4	4	3	-	11
	6	-	-	-	1	-	-	-	1
	7	-	-	2	1	6	1	-	10
	8	-	-	-	1	3	-	-	4
	**Total**			**2**	**7**	**13**	**4**		**26**
**IM, 500 oncospheres**	9	-	-	-	-	1	5	-	6
**IM, 1000 oncospheres**	10	-	-	-	-	1	4	-	5
**Total**				**2**	**15**	**31**	**13**		**61**

SA: subarachnoid. IM: intramuscular.

**Fig 3:**
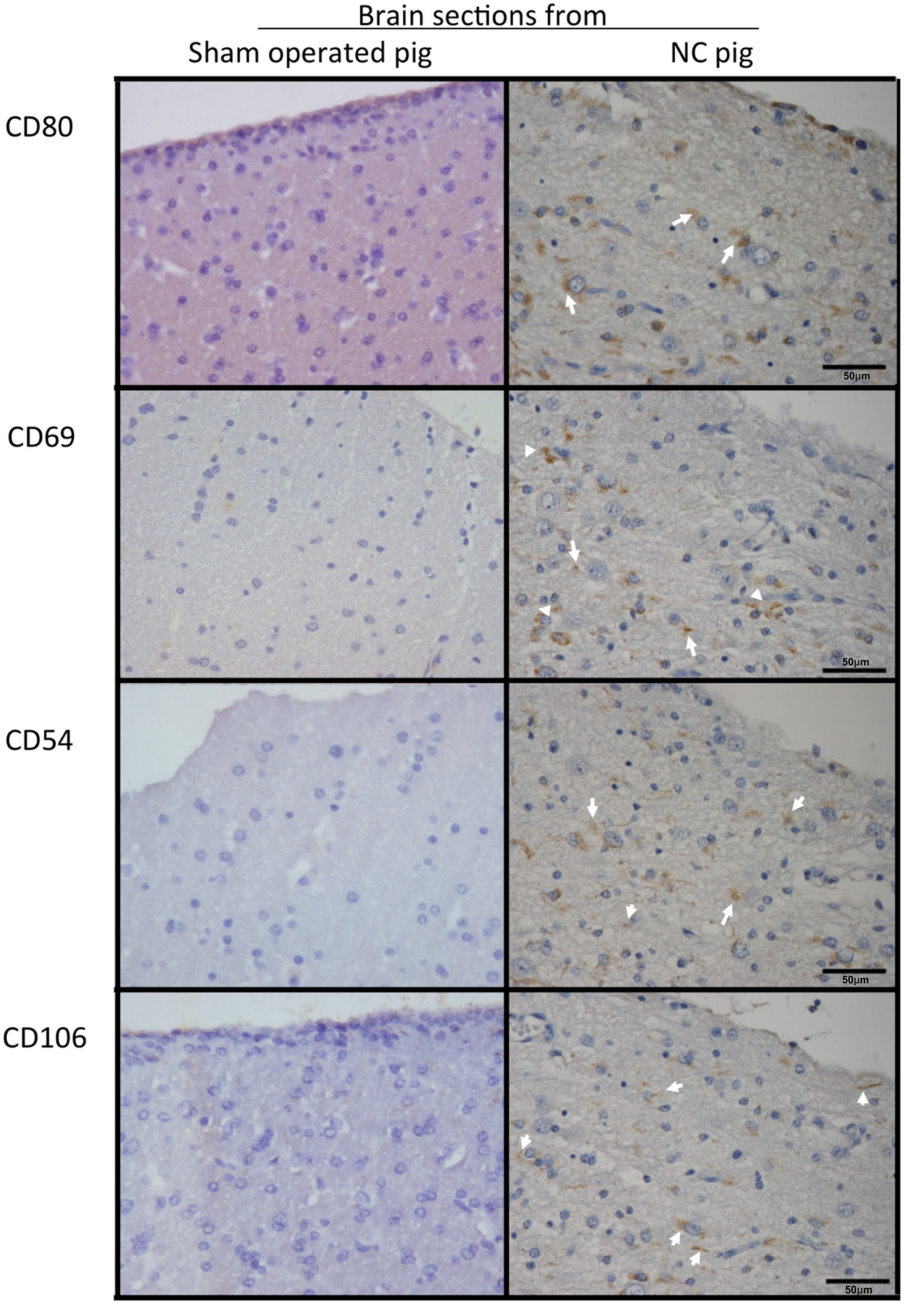
Expression of inflammatory cell markers in brain tissues. Six-micrometer sections of pig brain tissues proximal to installed parasites and sections of an equivalent region from sham-operated pigs (40X) were incubated with antibodies against inflammatory cell markers (CD106, CD80, CD54, and CD69). Non-immunized rabbit serum (control) showed no reaction. Arrows indicate regions where inflammatory markers are expressed. CD106 was expressed in brain microvessels, while CD80 was expressed in microglia cells.

**Fig 4:**
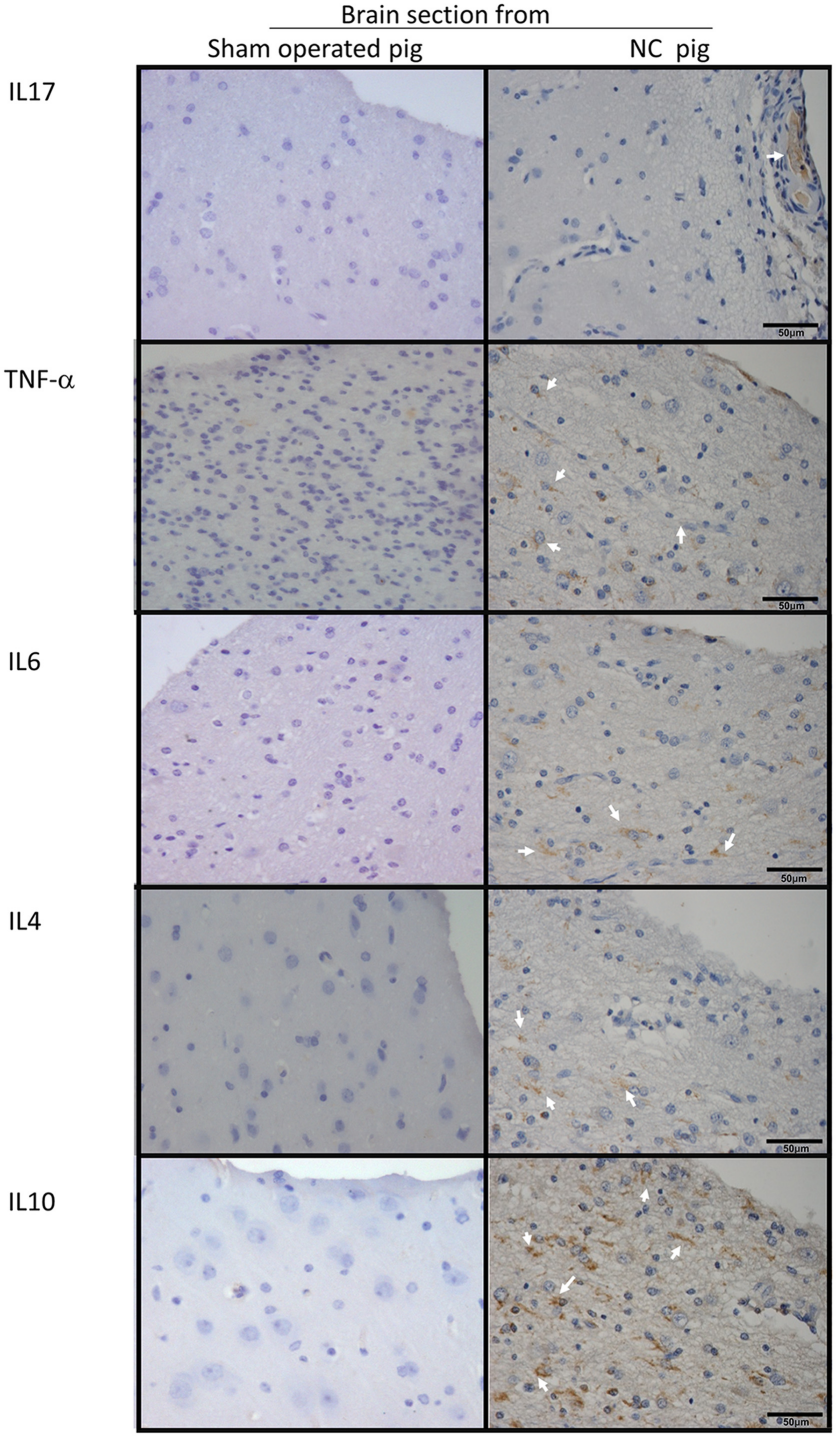
Expression of inflammatory and non-inflammatory cytokines. Six-micrometer sections of pig brain tissues proximal to installed parasites and sections of an equivalent region from sham-operated pigs (40X) were incubated with antibodies against inflammatory cytokines (IL-17, TNF-α, and IL6), the non-inflammatory cytokine IL-10 and a Th2-prototype cytokine. Arrows indicate regions where cytokines were expressed.

**Fig 5:**
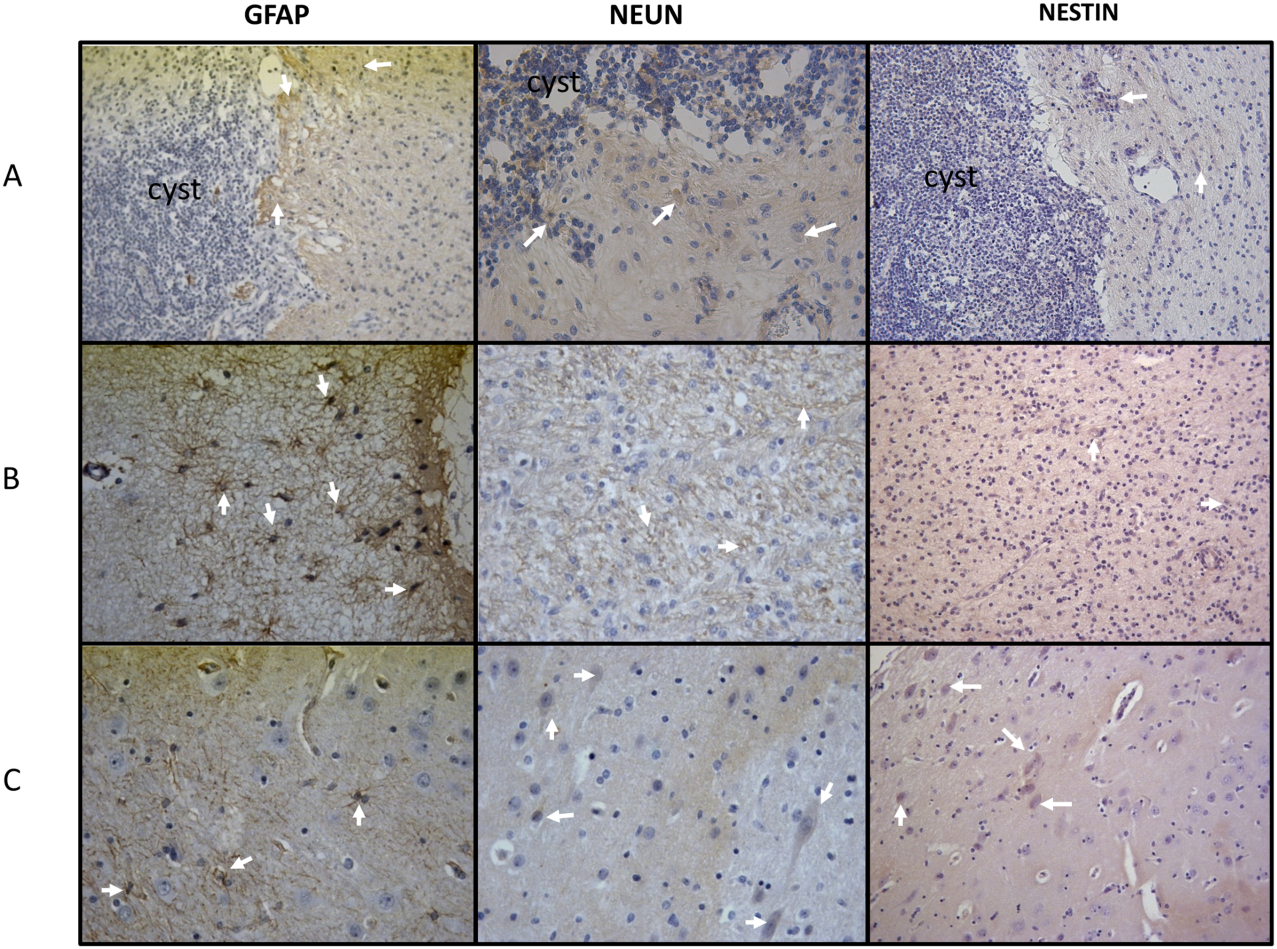
Expression of GFAP, NeuN, and nestin. (Line A) Parasite and brain tissue adjacent to it. (Line B) Tissue adjacent to the parasite. (Line C) Brain tissue distal to the parasite (40X).

**Fig 6:**
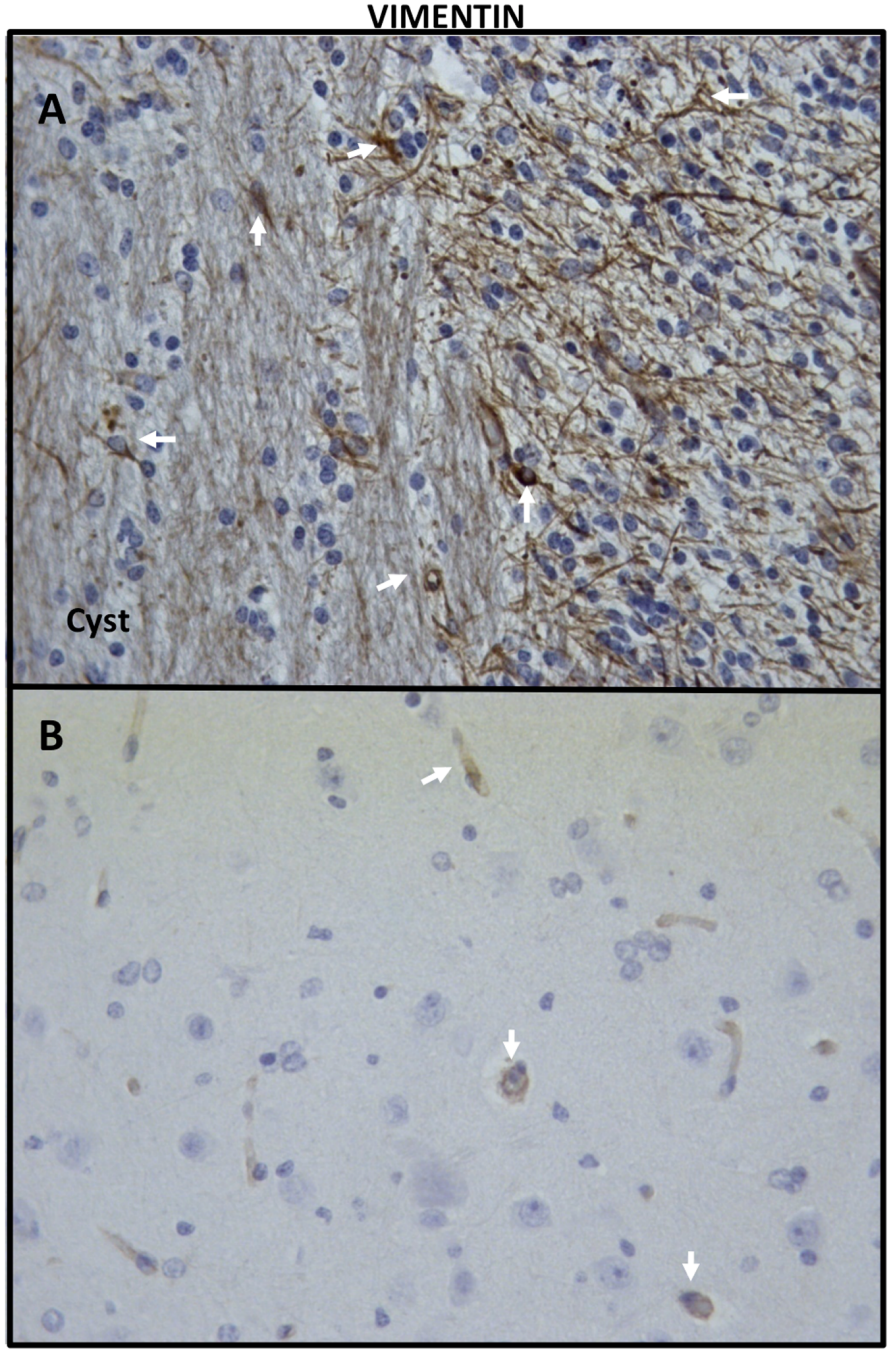
Expression of vimentin. The immunohistochemical analysis of vimentin expression showed that immature astrocytes were more frequently found in areas proximal to the parasite (A) than in tissues distal to the parasite (B) (40X).

The immunohistochemical analysis of the brain tissues surrounding cysticerci showed a high expression of CD80, CD106, CD69, and IL-10; a moderate expression of IL4, TNF-α, and CD54, and a low expression of IL-17 and IL-6 with respect to brain tissues from sham-operated pigs (Figs 3 and 4). GFAP and vimentin (indicators of immature astrocytes) were highly expressed in tissues proximal to the parasite, but expression lowered in tissues distal to the parasite (Figs 5 and 6). In general, the expression of NeuN (viable neurons) and nestin (immature neurons) was low, particularly in tissues proximal to the parasite, and increased slightly in tissues distal to the parasite (Fig 5).

**Fig 7:**
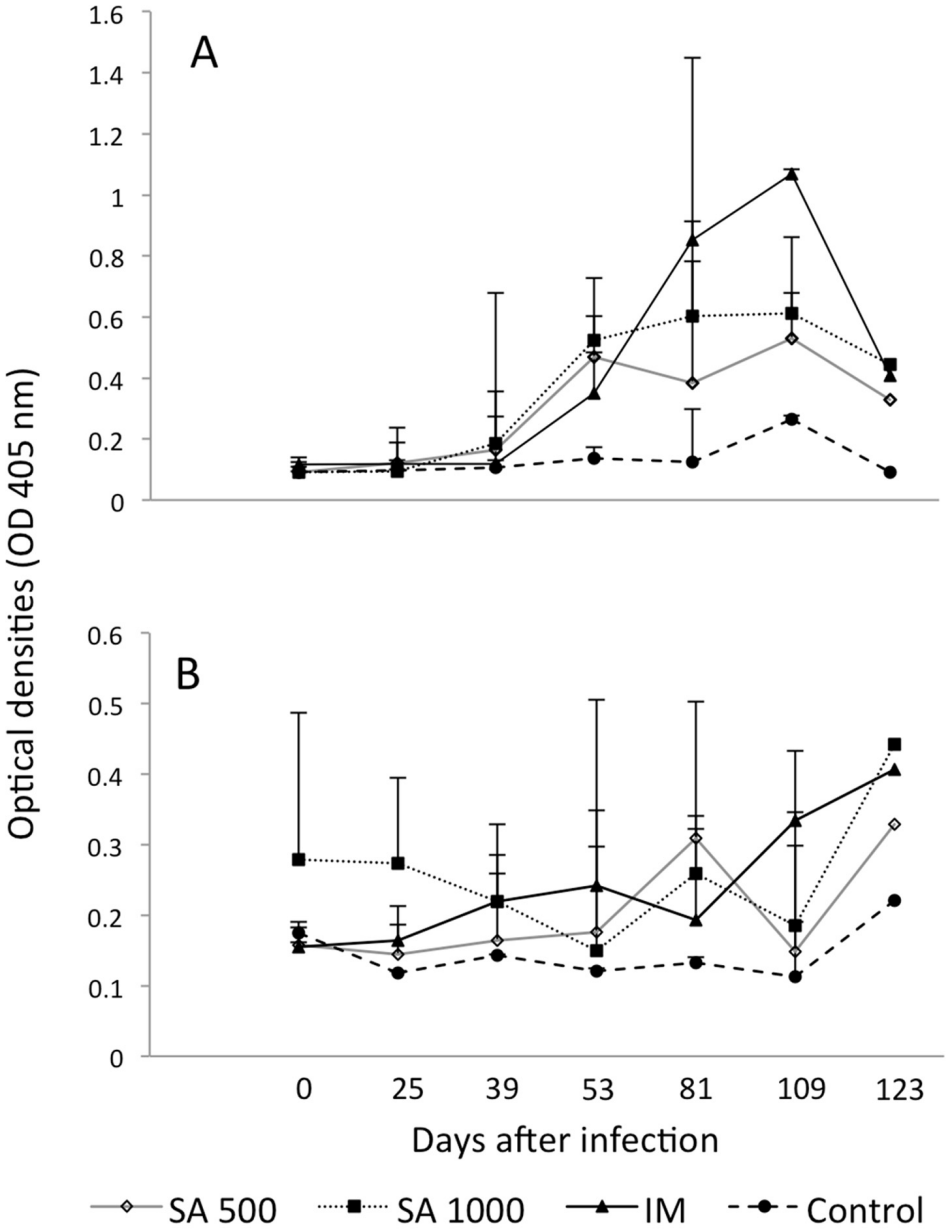
Kinetics of serum anti-cysticercal antibodies and HP10 secretion antigen levels after implantation of activated oncospheres in pigs. Two doses of activated oncospheres (500 or 1000) were surgically implanted in the subarachnoid space (SA) of the brain or in muscular tissues (IM). Two pigs were not infected (control). (A): Serum antibody levels. (B): Serum HP10 secretion antigen levels.

The level of anti-cysticercal antibodies and of the HP10 antigen in serum were measured at seven times during the experiment (Fig 7A and 7B, respectively). Samples were positive to antibodies at day 53 post-infection (PI) in all the animal groups (intramuscular and subarachnoid inoculation). Although a decrease was observed at the time of sacrifice, all sera remained positive during the experiment (Fig 7A). Antibody levels were significantly higher in the groups inoculated in the subarachnoid space (SA) (500 oncospheres, *R* = 0.75, *P* = 0.05; 1000 oncospheres, *R* = 0.80, *P* = 0.03), while only a tendency was observed in the group inoculated intramuscularly (IM) (*R* = 0.73, *P* = 0.06). The animals intramuscularly challenged exhibited higher antibody levels. Significant differences in OD values between groups of animals were observed at different times: at day 53 PI, control versus inoculation of 500 oncospheres in SA (*P* = 0.01); at day 81 PI, control versus IM inoculation (*P* = 0.02), controls versus inoculation of 1000 oncospheres in SA (*P* = 0.03), and inoculation of 500 oncospheres in SA versus IM (*P* = 0.02); at day 123 PI, control and IM inoculation (*P* = 0.001). Antibody levels did not increase in control pigs during the experiment (*R* = 0.48, *P* = 0.27).

With respect to HP10 serum levels (Fig 7B), while the three groups of pigs challenged with oncospheres were positive at the time of sacrifice, no significant increase was observed during the four months of the experiment (*R* = 0.62, *P* = 0.14; and *R* = 0.31, *P* = 0.49 when 500 and 1000 oncospheres were inoculated in SA respectively). Only in the IM inoculated group a significant increase was observed (*R* = 0.88, *P* = 0.008). OD values at each sampling time were not significantly different between groups.

## Discussion

The feasibility of infecting pigs by surgical implantation of activated oncospheres in the central nervous system is explored in this study.

Two individual experiments were performed, with important differences with regard to the efficiency of establishment. While only three parasites developed successfully in the first one, the implantation of multiple cysticerci in the central nervous system was observed in the second. These discordant results could be due to differences on the viability of the oncospheres used in each experiment. In the first experiment, the tapeworm was kept in refrigeration for 4 weeks before use, and only 5% of the oncospheres were activated when evaluated before implantation. In contrast, the tapeworm was kept for less than one week in refrigeration before use in the second experiment, and 85% of the oncospheres were activated before implantation. On the other hand, slight variations in the activation protocol of the oncospheres in both experiments could have had an impact on infection efficiency. For instance, it is likely that the trypsin added to pancreatin in the second experiment played a role in the higher oncosphere activation rate. These results point to the need of using oncospheres with high activation rates to achieve a higher infection efficiency. However, since both variables (the time elapsed between adult taenia recovery and infection, and the activation protocol) were changed in the same experiment, the main factor underlying these differences could not be determined in this work.

At necropsy time, 4 months after inoculation, most cysticerci were degenerating, with an exacerbated inflammatory reaction surrounding them. However, the proportion of vesicular cysticerci was significantly higher in the brain with respect to the cysts in muscle (*P* = 0.01). This observation is in accordance with previous works in naturally infected pigs [24,25]. It is likely that the higher destruction rates of muscle cysticerci are related to a more effective inflammatory response in the periphery. In contrast, the magnitude of the inmmunoinflammatory response is tightly regulated in the central nervous system, and cysticerci may remain for longer periods without damage [31]. However, most parasites located in the central nervous system were also in a degenerative stage. In humans, parasites reaching the brain are also mostly calcified at diagnosis, as shown in CT-scan-based epidemiological studies [32,33]. However, it is also known that brain cysticerci may persist vesicular for months or even years in humans [34]. It is thus probable that neurosurgery in our model generate additional inflammation, which could be promoting parasite degeneration. It is also possible that the hatching/activation process in the gastrointestinal track during natural infection confers some additional property to the oncospheres that will let them survive for longer periods in the central nervous system. Disregarding the factors involved in cysticercal destruction, this observation will be important to take into account when further experiments are performed to evaluate potential treatments. Indeed, the time elapsed between oncosphere implantation, treatment, and sacrifice should be shortened when comparing different therapeutic schemes.

In this study, we found that the infection efficiency was higher when lower doses of oncospheres were used in the challenge (3.6% using 1000 oncospheres and 5.4% when 500 oncospheres were inoculated, *P* = 0.1). On the other hand, the proportion of vesicular parasites was higher when higher oncospheres doses were implanted in the brain (44.4% versus 33.3%, *P* = 0.44). Similar tendencies were previously reported in a dose-response study performed in pigs [25]. These findings could reflect the fact that the immune mechanisms involved in the control of oncosphere establishment and in cysticerci destruction are different.

The inflammatory reaction surrounding most of the parasites was evident and most cysticerci were at grade 3 and 4 in the grading system by Aluja & Vargas [30]. Cells were mostly lymphocytes, plasma cells, macrophages, and eosinophils, a finding in accordance with the result of natural infections [35]. Immunohistochemical analysis showed a high lymphocyte activation, as revealed by the high expression of the CD69 activation marker and by the activation of astrocytes in parasite proximity. The high expression of CD106 (the vascular cell adhesion molecule VCAM-1) may result from the presence of TNF-α, which is known to increase the expression of adhesion molecules in endothelial cells, favoring the adhesion of peripheral leukocytes to enter the brain and therefore promoting brain inflammation [36]. It is noteworthy that the expression of proinflammatory cytokines (IL-6, IL-17) was not especially high, contrasting with the high expression of the regulatory cytokine IL-10. It is possible that regulatory factors secreted by the parasite accounted for this observation. Indeed, several molecules with potentially immunomodulatory functions have been found in the recently reported genome of *Taenia solium* [37]. The expression of the neuronal markers NeuN and nestin was low, although it increased slightly in brain tissues distal to the parasite with respect to tissues proximal to the parasite; this finding could reflect the neuronal damage in the cysticercus proximity. The high expression of mature and immature astrocyte markers in areas proximal to the parasite demonstrates the inflammatory reaction surrounding the parasite, which includes astrocyte activation.

Even though an assessment of the specificity of these changes was not the purpose of this work, it would be interesting to evaluate them by implanting other parasites in future experiments.

Serum specific anti-cysticercal antibodies increased in all groups of infected pigs, an additional result that demonstrates the close connection between the central and the peripheral immune system, as it has been extensively shown [38]. However, it is interesting to note that IM infection in experiment 2 elicited significantly higher antibody levels, than those observed in SA infected pigs at day 81 post-infection. The higher systemic antibody levels induced by peripheral infections may be due to the presence of multiple B cell follicles able to detect the presence of parasite antigens and promoting differentiation to plasmatic B cells, with the ensuing production of specific antibodies. On the other hand, the specific antibodies detected in the central nervous system may be produced in the periphery and then be centrally recruited due to some disruption in the blood brain barrier of cysticerci-infected pigs. The local intrathecal production of anti-cysticercal antibodies can also contribute to the detected central antibody levels.

None of the infected animals in experiment 2 exhibited neurological manifestations. This aspect, previously reported in other works [39] is noteworthy. In humans, several epidemiological studies have showed that an important proportion and in some cases most of the infected subjects are asymptomatic [32,33]. This same phenomenon could occur in pigs, and the fact that no neurological signs were detected could be due to the small number of pigs included in this study. However, it is interesting to note the high expression of the regulatory cytokine IL-10, while the frequency of IL-10-producing cells was relatively low [40]. Thus, it is possible that the absence of neurological symptoms in pigs be also due to a more active immunomodulatory process in this species. Disregarding the cause underlying the absence of clinical signs in pigs, this situation will not allow us to use this model to study the neurological aspects of the disease.

It should be noted that the model reproducibility was not evaluated in this study. Ten animals were used in the successful second experiment, and the issue of reproducibility should be addressed soon.

Finally, it is important to note the limitations of this model, mainly due to logistical requirements. Since adult pigs are not easy to manipulate, piglets were employed instead. This aspect is important, as our results could not reflect the infection in adult humans. Additionally, it has been shown that the clinical picture in human neurocysticercosis is quite different between children and adults [41]. On the other side, obtaining a *Taenia solium* tapeworm is not effortless, and the requirement of implanting eggs shortly after acquiring the taenia specimen limits its use to endemic countries. Furthermore, the model requires a technical team to care for animals after surgery, and the coordinated efforts of specialists (surgeons, veterinary personnel, parasitologists, immunologists, and medics) who might not be accustomed to work together. Nevertheless, we consider that all these difficulties need to be solved, since the results of studies using naturally infected pigs are often difficult to interpret, with the animals being not only infected in the central nervous system but also in the muscles, a fact that can change their immunological status.

In spite of its limitations, the new model for neurocysticercosis that we are proposing will allow for evaluating different therapeutic approaches that eventually could be employed to treat human neurocysticercosis.
